# To cage or to be caged? The cytotoxic species in ruthenium-based photoactivated chemotherapy is not always the metal†
†Electronic supplementary information (ESI) available: Synthetic procedures, singlet oxygen quantum yield, partition coefficient and cellular uptake measurements, cell culture and EC_50_ (photo)cytotoxicity assays. See DOI: 10.1039/c7cc03469e


**DOI:** 10.1039/c7cc03469e

**Published:** 2017-06-05

**Authors:** Jordi-Amat Cuello-Garibo, Michael S. Meijer, Sylvestre Bonnet

**Affiliations:** a Leiden Institute of Chemistry , University of Leiden , Einsteinweg 55 , 2333 CC , Leiden , The Netherlands . Email: bonnet@chem.leidenuniv.nl

## Abstract

In metal-based photoactivated chemotherapy (PACT), two photoproducts are generated by light-triggered photosubstitution of a metal-bound ligand: the free ligand itself and an aquated metal complex. By analogy with cisplatin, the aquated metal complex is usually presented as the biologically active species, as it can typically bind to DNA. In this work, we show that this qualitative assumption is not necessarily valid by comparing the biological activity, log *P*, and cellular uptake of three ruthenium-based PACT complexes: [Ru(bpy)_2_(dmbpy)]^2+^, [Ru(bpy)_2_(mtmp)]^2+^, and [Ru(Ph_2_phen)_2_(mtmp)]^2+^. For the first complex, the photoreleased dmbpy ligand is responsible for the observed phototoxicity, whereas the second complex is not phototoxic, and for the third complex it is the ruthenium bis-aqua photoproduct that is the sole cytotoxic species.

## Abstract 2

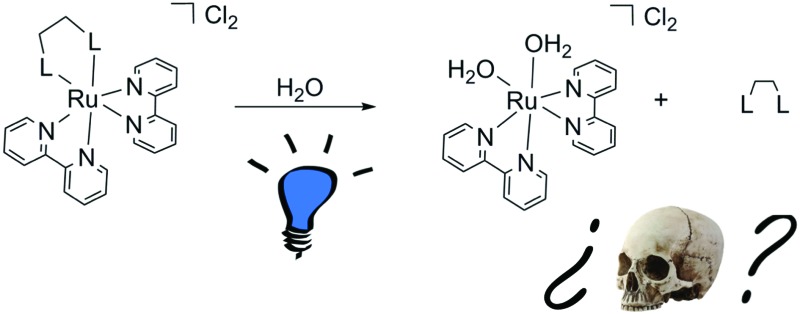
In metal-based photoactivated chemotherapy (PACT), two photoproducts are generated by light-triggered photosubstitution of a metal-bound ligand: the free ligand itself and an aquated metal complex.

## 

Ruthenium polypyridyl complexes are well known for their versatile and tunable photophysical and photochemical properties.^1^ In recent years, they have attracted much interest for molecular imaging and photopharmacology,^2^ and in particular for photodynamic Therapy (PDT) and photoactivated chemotherapy (PACT).^3^ In PACT like in PDT, a non-toxic or poorly cytotoxic prodrug becomes much more cytotoxic upon light irradiation, allowing for a time- and spatially-resolved delivery of the toxicity of the anticancer drug. However, whereas in PDT the photocytotoxicity relies on the photochemical generation of reactive oxygen species (ROS) such as singlet oxygen (^1^O_2_), in PACT a photochemical bond-breaking reaction occurs, which for coordination compounds is often realized *via* the photosubstitution of one of the ligands by water molecules.^4^ To make PACT ruthenium-based compounds, [Ru(bpy)_3_]^2+^-like complexes must be modified so that the triplet metal-centered excited states (^3^MC) come in close proximity to the triplet metal-to-ligand charge transfer states (^3^MLCT).^5^ Such modification typically entails the use of sterically hindered bidentate ligands such as 6,6′-dimethyl-2,2′-bipyridine (dmbpy) and its derivatives.^6^ For example, the irradiation of [Ru(bpy)_2_(dmbpy)]^2+^ in water (bpy = 2,2′-bipyridine) leads to the photosubstitution of dmbpy by two water molecules, generating the aquated species *cis*-[Ru(bpy)_2_(OH_2_)_2_]^2+^ (Scheme 1) that was shown to bind to plasmid DNA.^7^ When performed in the presence of growing cancer cells, this photoreaction clearly leads to photocytotoxicity, which many have interpreted as a consequence of the cytotoxicity of *cis*-[Ru(bpy)_2_(OH_2_)_2_]^2+^, by analogy to the cytotoxic aquated form of cisplatin, *cis*-[Pt(NH_3_)_2_(OH_2_)_2_]^2+^. On the other hand, many ruthenium polypyridyl complexes have been used as photocaging groups for neurotransmitters and organic enzyme inhibitors,^8^ for which the absence of acute toxicity is a pre-requisite. The parent compound [Ru(bpy)_2_Cl_2_], which thermally hydrolyzes into *cis*-[Ru(bpy)_2_(OH_2_)_2_]^2+^, was shown by the group of Reedijk not to be cytotoxic.^9^ As several groups have developed analogues of [Ru(bpy)_2_(dmbpy)]^2+^ for developing new PACT compounds, we asked ourselves which photoproduct, from the two that are formed upon light irradiation, actually is cytotoxic enough to kill cancer cells: the *cis* bis-aqua ruthenium complex or the free ligand?

**Scheme 1:**
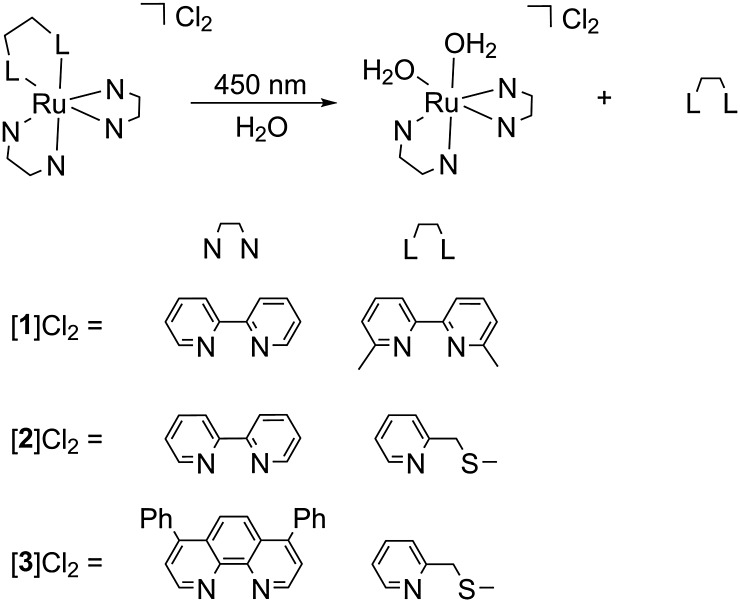
Chemical structures of PACT ruthenium compounds [**1**]Cl_2_–[**3**]Cl_2_ and their reaction upon blue light irradiation in water.

To address this question, we compared the known compound [Ru(bpy)_2_(dmbpy)]Cl_2_ ([**1**]Cl_2_) with a new photoactive compound [Ru(bpy)_2_(mtmp)]Cl_2_ ([**2**]Cl_2_) containing the bidentate chelate 2-methylthiomethylpyridine (mtmp).^7^ Thioethers are soft enough to coordinate well to ruthenium(ii) in the ground state, but they can be photosubstituted more efficiently than pyridines due to the relative weakness of the Ru–S bond in the excited state, compared to Ru–N bonds.^10^ Indeed when a solution of [**2**]Cl_2_ is irradiated with blue light (445 nm), a shift of the ^1^MLCT absorption maximum from 432 to 491 nm was observed, as well as two consecutive isosbestic points at 439 and 458 nm (Fig. 1a). Mass spectrometry after 50 minutes of irradiation (Fig. S1, ESI†) showed peaks at 140.2, 225.0, and 448.1 which corresponded to {mtmp + H}^+^ (calc. *m*/*z* = 140.2), [Ru(bpy)_2_(OH_2_)_2_]^2+^ (calc. *m*/*z* = 225.0), and [Ru(bpy)_2_(OH_2_)(OH)]^+^ (calc. *m*/*z* = 448.5), respectively. Thus, [**2**]^2+^ like [**1**]^2+^ leads upon light irradiation to the formation of the bis-aqua complex *cis*-[Ru(bpy)_2_(OH_2_)_2_]^2+^, but the free ligand obtained as the second photoproduct is mtmp, instead of dmbpy with [**1**]Cl_2_ (Scheme 1). The two sequential isosbestic points observed by UV-vis during the irradiation of [**2**]Cl_2_ suggest that photosubstitution takes place in a two-step process. The first process is very fast (it was completed within the first 30 seconds of irradiation) and is assumed to be the photosubstitution of one coordination bond of mtmp by a single water molecule. The second photosubstitution is much slower, as usually reported,^11^ and leads to the final photoproducts mtmp and *cis*-[Ru(bpy)_2_(OH_2_)_2_]^2+^. The quantum yield of this second process (*Φ*_PR_) was 0.0030 according to Glotaran global fitting (see the ESI†).

**Fig. 1:**
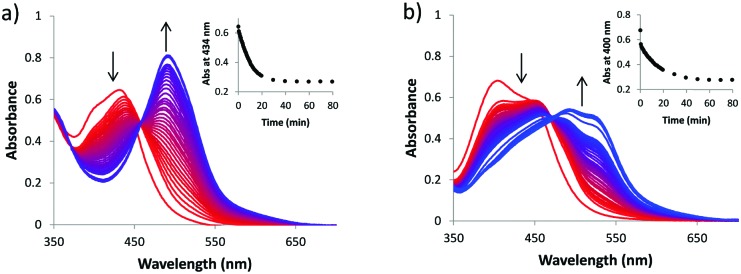
Evolution of the UV-vis absorption spectra of a solution of (a) [**2**]Cl_2_ and (b) [**3**]Cl_2_ in water upon irradiation with a 445 nm LED under N_2_ at 25 °C. Conditions: (a) 80 min, 0.109 mM, and 15.50 mW cm^–2^; (b) 80 min, 0.038 mM, and 13.65 mW cm^–2^.

The cytotoxicities of the free ligands dmbpy and mtmp were first compared in an A549 lung cancer cell line (adenocarcinomic human alveolar basal epithelial cells). Both organic ligands are rather lipophilic, as demonstrated by the octanol/water partition coefficient values (log *P*) of +3.29 and +1.63 for dmbpy and mtmp, respectively (Table 2). Both ligands are therefore expected to be taken up at least passively by the cells. The cell growth inhibition effective concentrations (EC_50_), *i.e.* the compound concentration at which the cell viability was reduced by 50% compared to the non-treated control, were measured following a protocol adapted from Hopkins *et al.* (see the ESI†).^12^ Clearly, dmbpy was found to be cytotoxic, with EC_50_ values of 8.7 and 6.5 μM in the dark and upon light irradiation, respectively (Fig. 2 and Table 1), whereas no cytotoxicity was observed for mtmp up to 200 μM. Although cellular localization of chemicals may differ whether they are simply incubated with the cells or generated inside the cells upon light irradiation of a prodrug such as [**1**]Cl_2_, this result suggests that the photocytotoxicity reported for [**1**]Cl_2_ may be at least partly due to the release of the dmbpy ligand.

**Fig. 2:**
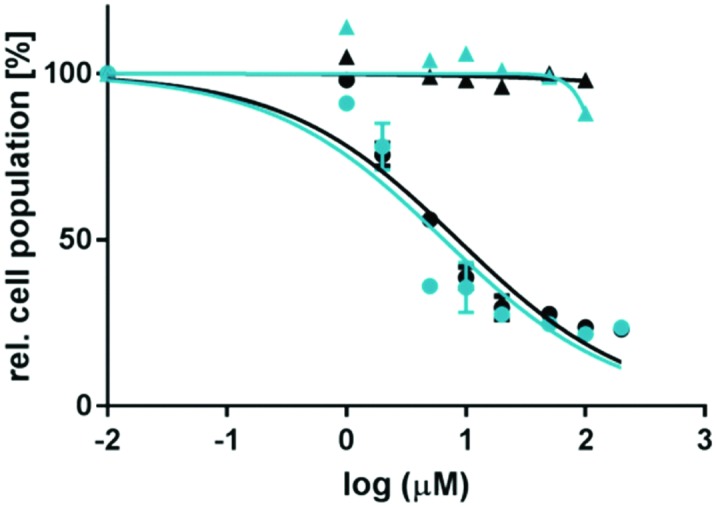
Dose–response curves for A549 cells incubated with dmbpy (circles) or mtmp (triangles) and irradiated for 10 min with blue light (455 nm, 6.5 J cm^–2^) 6 h after treatment (blue data points), or left in the dark (black data points). Phototoxicity assay outline: cells seeded at 5 × 103 cells per well at *t* = 0 h, treated with dmbpy or mtmp at *t* = 24 h, irradiated at *t* = 30 h, and SRB cell-counting assay performed at *t* = 96 h. Incubation conditions: 37 °C and 7% CO_2_.

**Table 1:** Cancer cell growth inhibition effective concentrations (EC_50_ values with 95% confidence interval in μM), in the dark and upon blue light irradiation (6.5 J cm^–2^), for [**1**]Cl_2_, [**2**]Cl_2_, [**3**]Cl_2_, dmbpy, and mtmp on lung cancer cells (A549); photoindices (PIs) defined as EC_50,dark_/EC_50,light_

	[**1**]Cl_2_	CI (95%)	[**2**]Cl_2_	CI (95%)	[**3**]Cl_2_	CI (95%)	dmbpy	CI (95%)	mtmp	CI (95%)
EC_50_ dark (μM)	210	–41	>150	—	2.66	–0.46	8.56	–2.76	>150	—
		+51		—		+0.56		+4.08		—
EC_50_ light (μM)	10.9	–4.3	>150	—	0.48	–0.08	6.55	–2.54	>150	—
		+7.1		—		+0.10		+4.17		—
PI	19		—		6		1.3		—	

In a second step, the EC_50_ values of complexes [**1**]Cl_2_ and [**2**]Cl_2_ were measured in A549 cells, both in the dark and upon blue light irradiation, and following the same protocol applied for the free ligand (Table 1). The selected light dose (6.5 J cm^–2^) guarantees that no toxic effect for the cells occurs due to the irradiation itself.^12^ At that light dose, both [**1**]Cl_2_ and [**2**]Cl_2_ are fully activated below 40 μM (see Fig. S4 and ESI†). As shown in Fig. 3, no significant decrease in the cell population was observed after treatment with less than 100 μM of complex [**1**]Cl_2_ or [**2**]Cl_2_ in the dark (Table 1). Thus, these species can be considered to be essentially non-cytotoxic in the dark. After blue light irradiation, an EC_50_ value of 10.9 μM was found for [**1**]Cl_2_, corresponding to a photoindex of 19, which qualitatively fits the data reported by Glazer *et al.* on this compound.^7^ However, no phototoxicity was observed for [**2**]Cl_2_, in spite of the fact that this compound also delivers the *cis*-[Ru(bpy)_2_(OH_2_)_2_]^2+^ species upon irradiation.

**Fig. 3:**
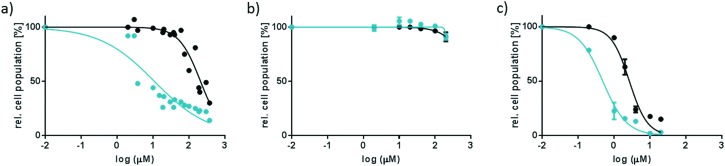
Dose–response curves for A549 cells incubated with [**1**]Cl_2_ (a), [**2**]Cl_2_ (b), or [**3**]Cl_2_ (c) and irradiated for 10 min with blue light (455 nm, 6.5 J cm^–2^) 6 h after treatment (blue data points), or left in the dark (black data points). Phototoxicity assay outline: cells seeded at 5 × 10^3^ cells per well at *t* = 0 h, treated with [**1**]Cl_2_, [**2**]Cl_2_, or [**3**]Cl_2_ at *t* = 24 h, irradiated at *t* = 30 h, and SRB assay performed at *t* = 96 h. Incubation conditions: 37 °C and 7% CO_2_.

In order to explain these differences, the octanol/water partition coefficient (log *P* value; see the ESI† ^^13), the cellular uptake, and the quantum yield for singlet oxygen generation were measured for both complexes (Table 2). log *P* values of –1.42 and –1.33 were found for [**1**]Cl_2_ and [**2**]Cl_2_, respectively, which means that both complexes have similar hydrophilicities and are not prone to enter the cell by passive diffusion through the membrane. As expected from these high hydrophilicity values, the cellular uptake before light activation, measured by ICP-MS by incubating A549 cells with [**1**]Cl_2_ or [**2**]Cl_2_ at 20 and 80 μM, respectively, for 6 h in the dark, was found to be very low: 1.32 and 1.27 ng of ruthenium were found per million cells for [**1**]Cl_2_ and [**2**]Cl_2_, respectively, compared to values usually found above 10–20 ng Ru per 10^6^ cells for compounds that are well taken up.^14^ Thus, the higher cytotoxicity found for [**1**]Cl_2_ after light activation cannot be attributed to a higher uptake of the complex prior to irradiation.

**Table 2:** Partition coefficient (log *P* values), singlet oxygen generation quantum yields (*Φ*_Δ_), and cellular uptake of [**1**]Cl_2_, [**2**]Cl_2_, [**3**]Cl_2_, dmbpy, and mtmp

	[**1**]Cl_2_	[**2**]Cl_2_	[**3**]Cl_2_	dmbpy	mtmp
log *P*	–1.42	–1.33	0.29	3.29^*a*^	1.63^*a*^
*Φ*_Δ_	0.023	<0.005	0.02	—	—
Cellular uptake (ng Ru × 10^6^ cells)	1.32 ± 0.06	1.27 ± 0.10	—	—	—

^*a*^log *P* estimation model from ChemDraw Professional (v16.0, CambridgeSoft).

Many published phototherapeutic ruthenium complexes are excellent PDT agents, *i.e.*, they generate ^1^O_2_*via* energy transfer from the ^3^MLCT to molecular oxygen present in the cells.^15^ Although it is commonly admitted that photosubstitutionally labile ruthenium complexes are poor singlet oxygen generators, the experimental values of ^1^O_2_ generation quantum yields (*Φ*_Δ_) are very rare in the literature for PACT compounds. In order to rule out that [**1**]Cl_2_ and [**2**]Cl_2_ may act as PDT agents, *Φ*_Δ_ values were experimentally determined for both complexes under blue light irradiation (450 nm), by direct detection of the 1274 nm infrared phosphorescence of ^1^O_2_ in CD_3_OD. *Φ*_Δ_ values of 0.023 and <0.005 were found for [**1**]Cl_2_ and [**2**]Cl_2_, respectively, using [Ru(bpy)_3_]Cl_2_ as a reference (*Φ*_Δ_ = 0.73).^16^ Thus, since both complexes are mediocre photosensitizers for ^1^O_2_, the phototoxicity of [**1**]Cl_2_ cannot be a photodynamic effect.

To summarize, [**1**]Cl_2_ and [**2**]Cl_2_ have similar negative log *P* values, similarly low cellular uptake after 6 h incubation in the dark, similarly low ^1^O_2_ generation quantum yields, and they both form [Ru(bpy)_2_(OH_2_)_2_]^2+^ upon light irradiation. Their main difference is that they photochemically release either dmbpy or mtmp, respectively. Meanwhile, we also demonstrated three points. First, the light activation of [**1**]Cl_2_ resulted in a 19-fold lower EC_50_ value compared to that obtained in the dark, whereas the light irradiation of [**2**]Cl_2_ does not influence an already negligible cytotoxicity. Second, dmbpy is cytotoxic to A549 cells, whereas mtmp is not. Third, the EC_50_ value of [**1**]Cl_2_ after irradiation (10.9 μM) is close, in the same protocol, to the EC_50_ value found for dmbpy (6.6 μM). Altogether, these results strongly suggest that the phototoxicity observed with complex [**1**]Cl_2_ is caused by the dmbpy ligand that is photoreleased and taken up after extra-cellular activation, rather than by the *cis*-[Ru(bpy)_2_(OH_2_)_2_]^2+^ species. In other words, [Ru(bpy)_2_(OH_2_)_2_]^2+^ is a photocaging group for the cytotoxic dmbpy ligand, rather than the reverse!

These surprising results do not, in our eyes, discredit the concept of ruthenium-based PACT. The problem of compounds such as [**1**]Cl_2_ or [**2**]Cl_2_ is only that their ruthenium-based photoproduct, *cis*-[Ru(bpy)_2_(OH_2_)_2_]^2+^, is not lipophilic enough to cross membranes and cause significant damage inside the cells. To demonstrate this idea, we synthesized a much more lipophilic version of compound [**2**]Cl_2_, *i.e.*, [Ru(Ph_2_phen)_2_(mtmp)]Cl_2_ ([**3**]Cl_2_, Ph_2_phen = 4,7-diphenyl-1,10-phenanthroline, see Scheme 1), by reacting [Ru(Ph_2_phen)_2_Cl_2_] with mtmp in ethylene glycol at 115 °C (see the ESI†). [**3**]Cl_2_ has a much higher log *P* value of 0.28, as expected from the more lipophilic Ph_2_phen spectator ligands. The photoreactivity of [**3**]Cl_2_ in water under blue light irradiation (445 nm) is similar to that of [**2**]Cl_2_: a shift of the ^1^MLCT absorption maximum from 404 to 492 nm and two sequential isosbestic points at 447 and 472 nm were observed (Fig. 1b). Mass spectrometry after 70 min of irradiation (Fig. S1b, ESI†) also showed photosubstitution of the non-toxic mtmp ligand, with peaks at 140.2, 412.3, and 424.5, corresponding to {mtmp + H}^+^, [Ru(Ph_2_phen)_2_(MeCN)(OH_2_)]^2+^ (calc. *m*/*z* = 412.6), and [Ru(Ph_2_phen)_2_(MeCN)_2_]^2+^ (calc. *m*/*z* = 424.1), respectively. The last two species are formed in the mass spectrometer and demonstrate the photochemical formation of the bis-aqua photoproduct [Ru(Ph_2_phen)_2_(OH_2_)_2_]^2+^. The photosubstitution has a quantum yield of 0.0010, slightly lower than that found for [**2**]Cl_2_, and the ^1^O_2_ generation quantum yield was similar to that found for [**1**]Cl_2_ (*i.e.*, *Φ*_Δ_ = 0.020; see Table 2). Thus, [**3**]Cl_2_ is a bad PDT sensitizer but a good PACT compound, as like [**2**]Cl_2_ it photosubstitutes the non-toxic mtmp ligand to deliver [Ru(Ph_2_phen)_2_(OH_2_)_2_]^2+^, a lipophilic analogue of [Ru(bpy)_2_(OH_2_)_2_]^2+^. In A549 cells, [**3**]Cl_2_ had a higher cytotoxicity in the dark (EC_50_ = 2.66 μM), as expected from its higher lipophilicity. Critically, the EC_50_ value decreased 6-fold down to 0.48 μM under a blue light dose of 6.5 J cm^–2^. Such increased cytotoxicity can, this time, only be attributed to the photochemical generation of [Ru(Ph_2_phen)_2_(OH_2_)_2_]^2+^, as the second photoproduct, mtmp, is non-toxic. [**3**]Cl_2_ is thus a true metal-based PACT compound where the toxicity of the Ru-based aqua species is “caged” *via* coordination of the mtmp ligand. Overall, our results demonstrate that determining which photoproduct is the cytotoxic species is not straightforward, as factors such as ligand toxicity, lipophilicity of the prodrug, cellular uptake and localization, and/or singlet oxygen generation may all influence the phototoxicity of a given compound. Although we have demonstrated here that the phototoxicity of [**1**]Cl_2_ is not due to the ruthenium-based photoproduct but because of the released dmbpy ligand, that of compound [**3**]Cl_2_ demonstrates that PACT compounds where the Ru photoproduct bears the toxic load can be made, provided its lipophilicity is high enough for the compound to enter the cell.

The European Research Council is acknowledged for a Starting Grant to S. B. The Dutch Organization for Scientific Research (NWO) is acknowledged for a VIDI grant to S. B. Prof. Elisabeth Bouwman is kindly acknowledged for scientific discussion and support. The COST actions CM1105 “Functional metal complexes that bind to biomolecules” is acknowledged for stimulating scientific discussion.

## Supplementary Material

Supplementary information
