# Supplementary material for: To cage or to be caged? The cytotoxic species in ruthenium-based photoactivated chemotherapy is not always the metal
Source: Chem Commun (Camb). 2017 Jun 5;53(50):6768–71. doi: 10.1039/c7cc03469e (PMC5708332; doi:10.1039/c7cc03469e)
Supplement: Supplementary file 1 [file CC-053-C7CC03469E-s001.pdf]

Electronic Supplementary Material (ESI) for ChemComm.
This journal is © The Royal Society of Chemistry 2017

To cage or to be caged? The Cytotoxic Species in Ruthenium-Based PhotoActivated ChemoTherapy is Not Always the Metal

Jordi-Amat Cuello-Garibo,[a] Michael Meijer,[a] Sylvestre Bonnet[a]*
Email: bonnet@chem.leidenuniv.nl

[a] *Leiden Institute of Chemistry, Universiteit Leiden, Einsteinweg 55 2333 CC, Leiden, Netherlands*

Supplementary Information

Synthesis 2
Photochemistry 2
  Irradiation experiments followed by MS, UV-Vis 2
  Blue light irradiation in the cell irradiation setup 5
Singlet Oxygen quantum yield measurement 6
Partition coefficient (logP) 8
Cell culture and EC_{50} (photo)cytotoxicity assay 8
  General 8
  Cell culture 8
  Cell-irradiation setup 9
  Cytotoxicity assay 9
Cell uptake 10
References 11

Synthesis

The ligands 6,6'-dimethyl-2,2'-bipyridine and 4,7-Diphenyl-1,10-phenanthroline were purchased from Sigma-Aldrich, as well as cis-Bis(2,2′-bipyridine)dichlororuthenium(II) hydrate (cis-Ru(bpy)$_2$Cl$_2$). Lithium chloride (LiCl) was purchased from Alfa-Aesar and RuCl$_3$·3H$_2$O was provided by Prof. Dr. E. Bouwman. All reactants and solvents were used without further purification. The synthesis of cis-Ru(Ph$_2$phen)$_2$Cl$_2$, [**1**]Cl$_2$, [**2**]Cl$_2$, and the ligand 2-methylthiomethylpyridine was carried out according to literature procedures.[1-3]

Electrospray mass spectra (ESI-MS) were recorded by using a Thermoquest Finnagen AQA Spectrometer and a MSQ Plus Spectrometer. UV-Vis experiments were performed on a Cary Varian spectrometer. All ^{1}H NMR spectra were recorded on a Bruker DMX-400 spectrometer. Chemical shifts are indicated in ppm relative to the residual solvent peak.

[Ru(ph$_2$phen)$_2$(mtmp)]Cl$_2$ ([**3**]Cl$_2$). cis-Ru(Ph$_2$phen)$_2$Cl$_2$ (50 mg, 0.060 mmol) was dissolved in ethylene glycol (4 mL), after which mtmp (26 mg, 0.187 mmol) and Et$_3$N (28 µL, 0.200 mmol) were added. The reaction mixture was placed under N$_2$ atmosphere, deoxygenated, and heated at 115 ºC for 2 h. Then, the crude was purified by column chromatography on deactivated alumina using DCM as an eluent. The orange fraction was collected and the solvent was removed by rotatory evaporation. Traces of ethylene glycol were removed by co-evaporation with toluene. A dark orange solid was obtained after re-crystallization in Et$_2$O (30 mg, 50%). ^{1}H NMR (400 MHz, Methanol-*d*$_4$) δ 9.93 (d, *J* = 5.4 Hz, 1H), 8.80 (d, *J* = 5.4 Hz, 1H), 8.38 – 8.32 (m, 2H), 8.30 – 8.22 (m, 2H), 8.16 (d, *J* = 5.5 Hz, 1H), 8.13 (d, *J* = 5.1 Hz, 3H), 7.93 – 7.88 (m, 2H), 7.86 – 7.81 (m, 2H), 7.80 – 7.75 (m, 2H), 7.75 – 7.64 (m, 7H), 7.63 – 7.53 (m, 12H), 7.20 – 7.13 (m, 1H), 5.08 (d, *J* = 16.7 Hz, 1H), 4.61 (d, *J* = 16.5 Hz, 1H), 1.55 (s, 3H). ^{13}C NMR (101 MHz, MeOD) δ 154.09, 153.78, 153.50, 153.32, 152.31, 151.74, 151.59, 151.52, 150.91, 150.37, 150.11, 149.69, 149.23, 139.16, 137.07, 137.04, 136.93, 136.85, 131.21-130.24 (20C, 4 phenyl groups), 130.60,128.17, 127.84, 127.74, 127.65, 127.57, 127.36, 127.32, 127.14, 126.43, 126.00, 16.88. ESI MS m/z (calc): 452.8 (452.6 [**3**]$^{2+}$). UV-vis λ in nm (ε in M^{-1}.cm^{-1}): 405 (17281) in water.

Photochemistry

Irradiation experiments followed by MS, UV-Vis

UV-Vis spectroscopy was performed using a Cary Varian spectrometer equipped with temperature control set to 298 K and a magnetic stirrer. For the irradiation, a LED light source (λ_{ex} = 445 nm, with a Full Width at Half Maximum of 22 nm) with a light intensity of 15.50 or 13.65 mW·cm^{-2} (for [**2**]Cl$_2$ and [**3**]Cl$_2$ respectively) was used. Experiments were performed in a quartz cuvette containing 3 mL of solution. A stock solution of the desired complex was prepared using demineralized water, which was then diluted in the cuvette to the desired working concentration. When the experiment was carried under N$_2$ the sample was deoxygenated for 15 min by gentle bubbling of N$_2$ and the atmosphere was kept inert during the experiment by a gentle flow of nitrogen on top of the cuvette. A UV-Vis spectrum was measured every 30 s for the first 10 min, every 1 min for the next 10 min, and eventually every 10 min until the end of the experiment. Data was analysed with Microsoft Excel. The quantum yields of the photoreactions (Φ_{PR}) were calculated by modelling the time evolution of the absorbance spectrum of the solution using the Glotaran software (see Figure S2-S2). Experimental conditions are detailed in Table S1.

Table S1. Conditions of the photoreactions followed by UV-Vis and Mass spectrometry.

Complex	Stock solution			Working solution (mM)	Power 450 nm LED ($mW{\cdot}cm^{-2}$)
	w (mg)	V (mL)	M (mM)		
[**2**]Cl_2	1.0	10	0.164	0.109	15.50
[**3**]Cl_2	1.1	10	0.113	0.038	13.65

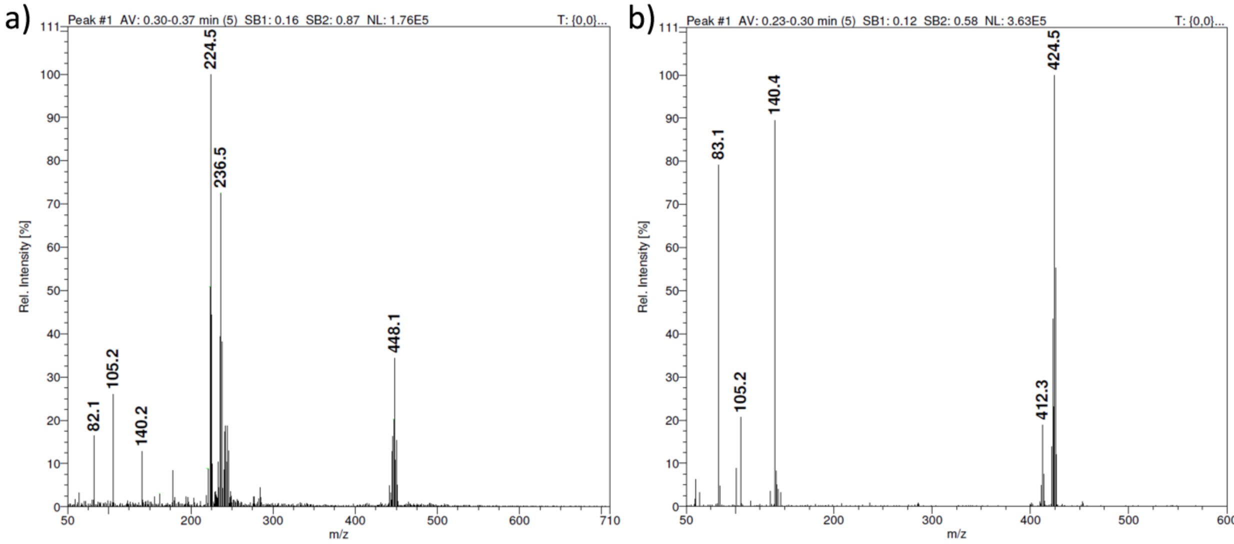

Figure S1. Mass spectrum of water solutions of (a) [2]Cl_2 and (b) [3]Cl_2 after 80 min irradiation with a 445 nm LED. a) Peaks corresponding to $\{mtmp+H\}^+$ (calcd *m/z* = 140.2), $[Ru(bpy)_2(OH_2)_2]^{2+}$ (calcd m/z = 225.0), $[Ru(bpy)_2(OH_2)(OH)]^+$ (calcd m/z = 448.5). b) Peaks corresponding to $\{mtmp + H\}^+$ (calcd m/z = 140.2) and $[Ru(Ph_2phen)_2(MeCN)_2]^{2+}$ (calcd m/z = 424.1). Conditions are detailed in Table S1.

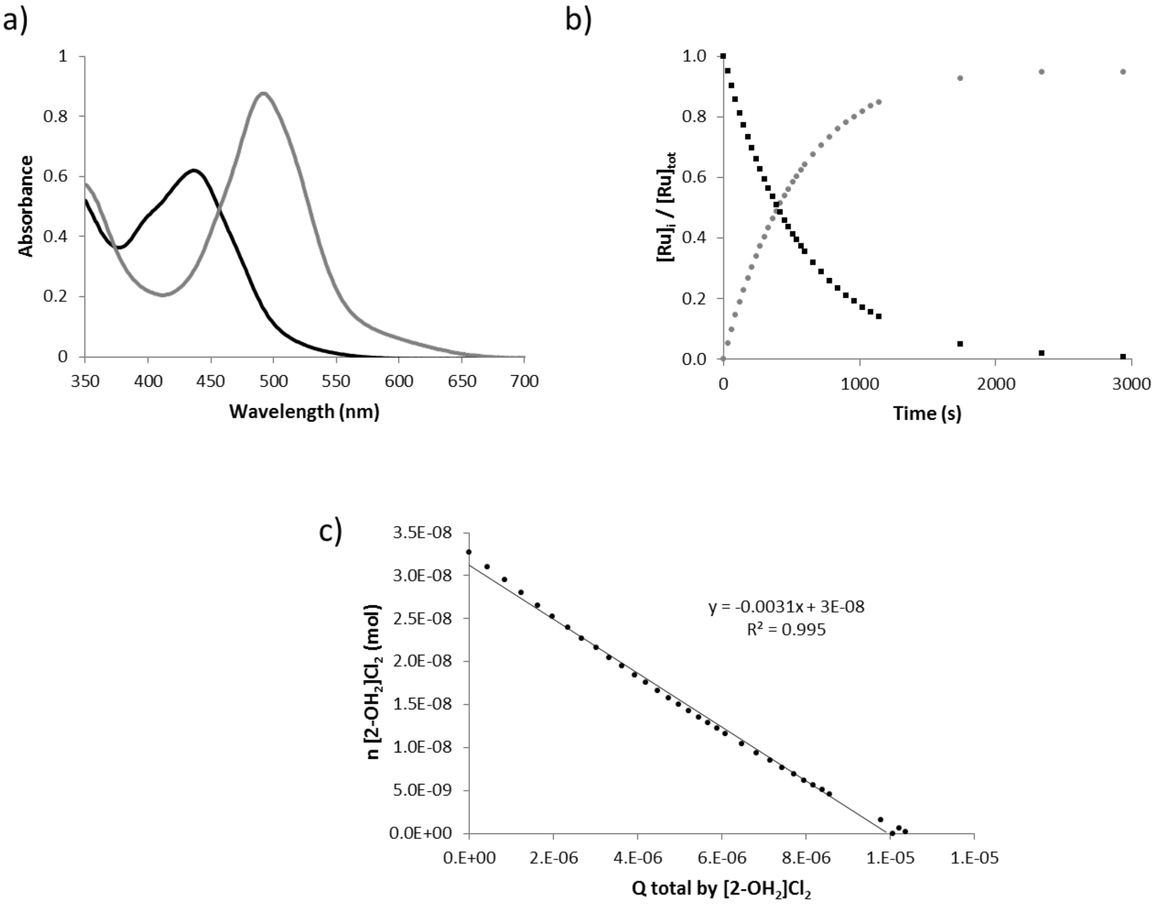

Figure S2. Kinetic data for the second step of the photosubstitution of [2]Cl_2 in water under N_2. a) Globally fitted absorption spectra of the mono-aqua intermediate [2-OH_2]Cl_2 (black) and [Ru(bpy)$_2$(OH_2)$_2$]Cl_2 (grey) according to modeling using the Glotaran software. b) Modelled evolution of the relative concentrations of [2-OH_2]Cl_2 (squares) and [Ru(bpy)$_2$(OH_2)$_2$]Cl_2 (circles) vs. irradiation time according to global fitting using Glotaran. c) Plot of the amount of [2-OH_2]Cl_2 (mol) vs. total amount of photons absorbed by [2-OH_2]Cl_2 (mol). The negative slope of the curve is the quantum yield of the formation of the bis-aqua complex. Conditions: 0.109 mM solution of [2-OH_2]Cl_2 in deminerilized water irradiated at 298 K under N_2 using a 445 nm LED at 15.50 mW·cm^{-2}.

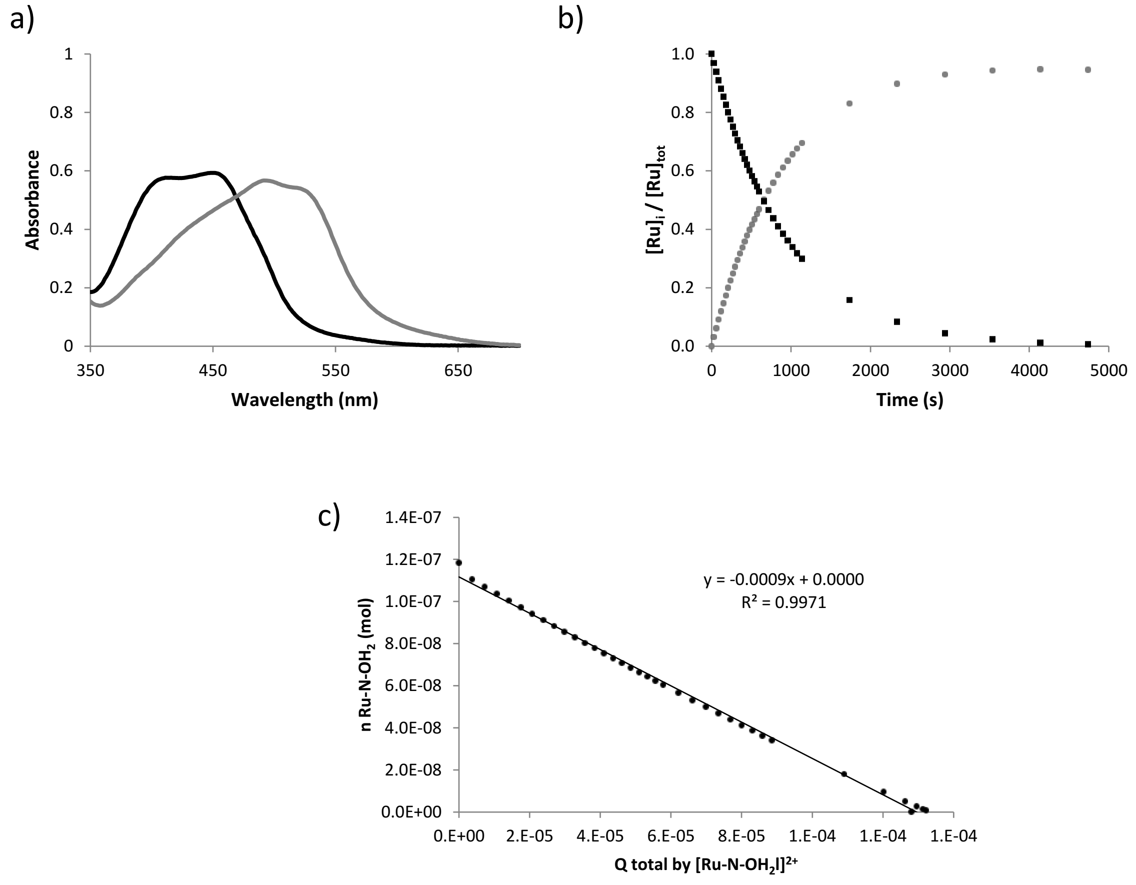

Figure S3. Kinetic data for the second step of the photosubstitution of [2]Cl_2 in water under N_2. a) Globally fitted absorption spectra of the mono-aqua intermediate[Ru(Ph_2phen)$_2$(η^1-mtmp)(OH_2)]Cl_2 ([3-OH_2]Cl_2 (black) and [Ru(Ph_2phen)$_2$(OH_2)$_2$]Cl_2 (grey) according to modeling using the Glotaran software. b) Modelled evolution of the relative concentrations of [3-OH_2]Cl_2 (squares) and [Ru(Ph_2phen)$_2$(OH_2)$_2$]Cl_2 (circles) vs. irradiation time according to global fitting using Glotaran. c) Plot of the amount of [3-OH_2]Cl_2 (mol) vs. total amount of photons absorbed by [3-OH_2]Cl_2 (mol). The negative slope of the curve is the quantum yield of the formation of the bis-aqua complex. Conditions: 0.038 mM solution of [3-OH_2]Cl_2 in deminerilized water irradiated at 298 K under N_2 using a 445 nm LED at 13.65 mW·cm^{-2}.

Blue light irradiation in the cell irradiation setup: which dose is necessary?

In order to assess which light dose should be used for photocytotoxicity assay, the photochemical reactivity of [**1**]Cl_2 and [**2**]Cl_2 was measured in 96-well plates, i.e. in the conditions of the cell experiments, but without cells and using UV-vis spectroscopy to measure to which extent the compounds are activated at different light doses. Two solution of each compound were prepared in Opti-MEM complete (40 µM and 200 µM) and distributed in a 96-well plate. The plate was irradiated with blue light (454 nm) at different irradiation times (0, 2, 5, 8, 10 min) using the blue LED source described in details in Hopkins *et al.*[6] At 40 µM and below both complexes received enough light at 10 min irradiation (dose 6.5 J.cm^{-2}) to be fully activated. At 200 µM complex [**2**]Cl_2 was only partly activated (Figure S4). Higher light doses would be necessary to fully activate the highest concentrations used for [**2**]Cl_2, but they would also be inherently cytotoxic to A549 cells, as described in Hopkins et al.[6] Thus, 10 minutes irradiation, for a dose of 6.5 J.cm^{-2}, was chosen for all photocytotoxicity experiments.

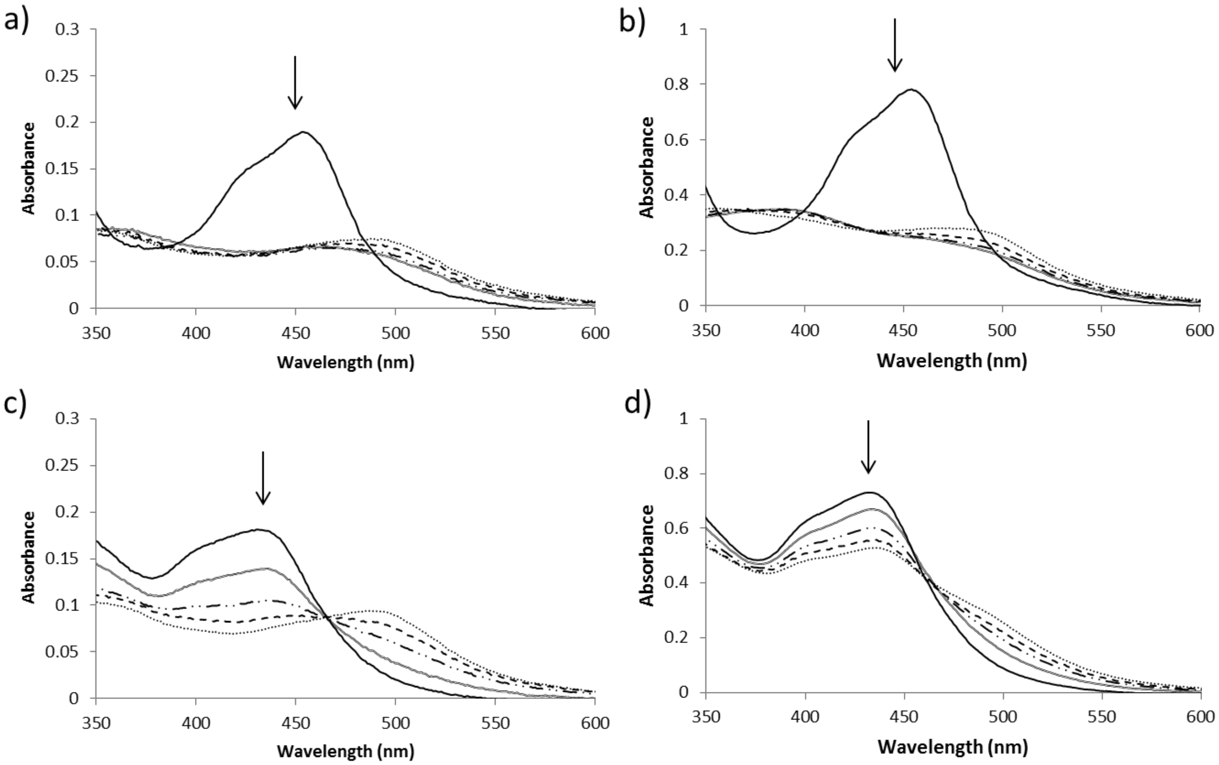

Figure S4. Evolution of the UV-vis spectrum of a well in a 96-well plate containing compound (a) [1]Cl_2 (40 µM), (b) [1]Cl_2 (200 µM), (c) [2]Cl_2 (40 µM), and (d) [2]Cl_2 (200 µM) in Opti-MEM complete, under blue light irradiation (37 °C) at 0 min (—), 2 min (=), 5 min (—·· —), 8 min (- - -), 10 min (···). In such conditions, 10 min irradiation correspond to a light dose of 6.5 J.cm^{-2}.

Singlet Oxygen quantum yield measurement

The quantum yield of singlet oxygen generation was determined in a custom-built setup (Figure S5), in which both UV-Vis absorption and infrared emission spectroscopy could be performed. All optical parts were connected with optical fibers from Avantes (Apeldoorn, The Netherlands), with a diameter of 200-600 µm. For each measurement, 500 µL of sample, consisting of the compound in deuterated methanol ($A_{450} \leq 0.1$ for 4.0 mm pathlength), was placed in a stirred 104F-OS semi-micro fluorescence cuvette (Hellma Analytics, Müllheim, Germany) in a CUV-UV/VIS-TC temperature-controlled cuvette holder from Avantes. The sample was allowed to equilibrate at 293 K for 5 minutes. Emission spectroscopy was performed with a 450 nm fiber-coupled laser (Laser system LRD-0450; Laserglow, Toronto, Canada), at 50 mW optical power (4 mm beam diameter; 0.4 W.cm^{-2}) at a 90° angle with respect to the spectrometer. The excitation power was measured using a S310C thermal sensor connected to a PM100USB power meter (Thorlabs, Dachau, Germany). Infrared emission spectra were measured from 1000 nm to 1400 nm using an Avantes NIR256-1.7TEC spectrometer, The infrared emission spectrum was acquired within 9 seconds, after which the laser was turned off directly. UV-Vis absorption spectra before and after emission spectroscopy were measured using an Avalight-DHc halogen-deuterium lamp (Avantes) as light source (turned off during emission spectroscopy) and an Avantes 2048L StarLine UV-Vis spectrometer as detector, both connected to the cuvette holder at a 180° angle. No difference in UV-Vis absorption spectrum was found due to exposure to the blue laser, showing that the singlet oxygen emission is that of the starting compound. All spectra were recorded with Avasoft 8.5 software from Avantes and further processed with Microsoft Office Excel 2010 and Origin Pro 9.1 software.

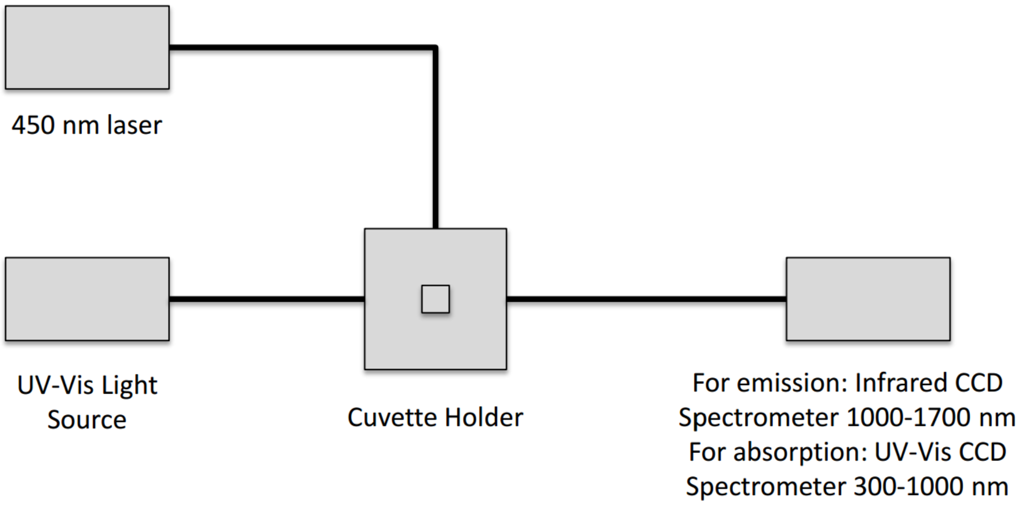

Figure S5. Setup for $^1O_2$ quantum yield measurement.

The quantum yield of singlet oxygen production was calculated using the relative method with $[Ru(bpy)_3]Cl_2$ as the standard (0.73 in CD_3OD)[4], according to Equation 1:

$$\Phi_{\Delta,sam} = \Phi_{\Delta,std} \times \frac{A_{450,std}}{A_{450,sam}} \times \frac{E_{sam}}{E_{std}}$$

Equation 1

where Φ_Δ is the quantum yield of singlet oxygen generation, A_{450} is the absorbance at 450 nm, E is the integrated emission peak of singlet oxygen at 1274 nm, and *sam* and *std* denote the sample and standard, respectively.

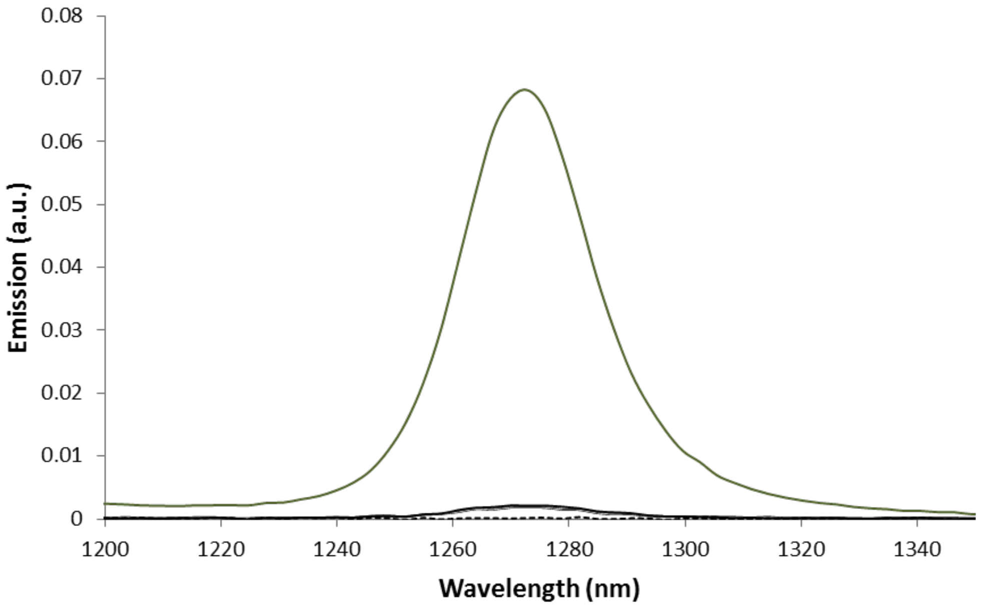

Figure S6. Time-integrated emission spectra of $[Ru(bpy)_3]Cl_2$ (green), $[1]Cl_2$ (black solid), $[2]Cl_2$ (black dashed), and $[3]Cl_2$ (black double line), irradiated with blue light (450 nm, 50 mW, 0.4 W.cm^2), stirred under air in CD_3OD at 298 K. Emission was measured over 9 s.

Partition coefficient (logP)

The partition coefficient determination was adapted from Wang *et al.*[5] Stock solutions of [**1**]Cl_2, [**2**]Cl_2, and [**3**]Cl_2 were prepared in octanol-saturated water (1 mM). Aliquots of the stock solutions (0.2 mL) were transferred per triplicate to 15 mL centrifuge tubes and diluted up to 1 mL with octanol-saturated water to give 0.2 mM solutions. Then, 1 mL of water-saturated octanol was added and the mixtures were shaken in a IKA Vibrax shaker for 1 h at 2200 rpm. Then, the mixtures were centrifuged (4300 rpm, 10 min, room temperature). Aliquots of the water layer (0.2 mL) were diluted with MilliQ water (2.4 mL) and 65% HNO_3 (0.4 mL) per duplicate, to give a final solution at 5% HNO_3. The ruthenium content of these samples was determined by ICP-OES using a Vista-MPX CCD Simultaneous ICP-OES. The partition coefficient values can be found in Table 2 and were determined by using Equation 2,

$$\log P_{oct} = \log \frac{[Ru]_{total} - [Ru]_{aq}}{[Ru]_{aq}}$$

Equation 2

where $[Ru]_{total}$ is the concentration of Ru in the control sample (where no water-saturated octanol was added) and $[Ru]_{aq}$ is the concentration of Ru in the aqueous layer as a mean of the six replicates.

Cell culture and EC_{50} (photo)cytotoxicity assay

General

Human cancer cell line A549 (human lung carcinoma) was distributed by the European Collection of Cell Cultures (ECACC), and purchased from Sigma Aldrich. Dulbecco's Modified Eagle Medium (DMEM,with and without phenol red, without glutamine), Glutamine-S(GM;200 mm), trichloroacetic acid (TCA), glacial acetic acid, sulfo-rhodamine B (SRB), and tris(hydroxylmethyl)aminomethane (Trisbase) were purchased from Sigma Aldrich. Fetal calf serum (FCS) was purchased from Hyclone. Penicillin and streptomycin were purchased from Duchefa and were diluted to a 100 mg/mL penicillin/streptomycin solution (P/S). Trypsin and Opti-MEM (without phenol red) were purchased from Gibco Life Technologies. Trypan blue (0.4 % in 0.81% sodium chloride and 0.06 % potassium phosphate dibasic solution) was purchased from BioRad. Plastic disposable flasks and 96-well plates were purchased from Sarstedt. Cells were counted by using a BioRad TC10 automated cell counter with Biorad cell-counting slides. UV/Vis measurements for analysis of 96-well plates were performed with a M1000 Tecan Reader. Cells were inspected with an Olympus IX81 microscope.

Cell culture

Cells were cultured in Dulbecco's Modified Eagle Medium containing phenol red, supplemented with 8.0% v/v fetal calf serum (FCS), 0.2% v/v penicillin/streptomycin and 0.9% v/v glutamax. Cell were incubated at 37 ºC at 7.0% CO_2 in 75 cm^2 T-flask and splitted once a week at 80-90% confluency. Cell were cultured for a maxium of 8 weeks for all biological experiment, and passaged at least twice after being thawed.

Cell-irradiation setup

The cell-irradiation system consisted of a Ditabis thermostat (980923001) fitted with two flat-bottomed micro-plate thermoblocks (800010600) and a 96-LED array fitted to a standard 96-well plate. The 454 nm LED (OVL-3324), fans (40 mm,24 VDC, 9714839), and power supply (EA-PS 2042-06B) were obtained from Farnell. See Hopkins *et al.* for a full description of the cell-irradiation setup.[6]

Cytotoxicity assay

Cells were seeded at t = 0 in 96-well plates at a density of 5000 cells/well in Opti-MEM supplemented with 2.4% v/v FCS, 0.2% v/v P/S, and 1.0% v/v glutamax (hereafter called Opti-MEM complete) (100 µL) and incubated for 24 h at 37 ºC and 7 % CO_2. After this period, aliquots (100 µL) of six different concentrations (2–200 µM for all the compounds, except for [**3**]Cl_2 where 0.1-20 µM were used) of freshly prepared stock solutions of [**1**]Cl_2, [**2**]Cl_2, [**3**]Cl_2, dmbpy, and mtmp in Opti-MEM were added to the wells in triplicate. Sterilized dimethylsulfoxyde (DMSO) was used to dissolve the compounds in such amounts that the maximum v/v% of DMSO per well did not exceed 0.5% v/v%. For every irradiated plate a parallel control plate was prepared and treated identically to the irradiated plate, but without irradiation. Plates were incubated in the dark for an additional 6 h. After this period, half of the plates were irradiated for 10 min with blue light (λ = 454 ± 11 nm, power density = 10.5 ± 0.7 mW cm^{-2}, irradiation time = 10 min, light dose = 6.5 Jcm^{-2}) and the other half were kept in the dark. After irradiation all the plates were incubated for an additional 66 h (making a total assay of 96 h) The cells were fixated by adding cold TCA (10 % w/v; 100 µL) in each well and the plates were stored at 4 ºC for at least 4 h as part of the sulforhodamine B (SRB) assay that was adapted from Vichai *et al.*[7] In short, after fixation TCA medium mixture was removed from the wells, rinsed with demineralized water three times and air dried. Then, each well was stained with 100 µL SRB (0.6 % w/v in 1% v/v acetic acid) for 30 min, the SRB was removed by washing with acetic acid (1 % v/v), and air dried. The SRB dye was solubilized with Tris base (10 mM; 200 µL), and the absorbance in each well was read at λ = 510 nm by using a M1000Tecan Reader.

The SRB absorbance data per compound per concentration were averaged over three identical wells (technical replicates, n_t = 3) in Excel and made suitable for use in GraphPad Prism. Relative cell populations were calculated by dividing the aver-age absorbance of the treated wells by the average absorbance of the untreated wells. In any case it was checked that the cell viability of the untreated cells of the samples irradiated were similar (maximum difference of 10%) to the unirradiated samples to make sure no harm was done by the light. The data from three independent biological replications was plotted versus log(concentration) [µm]. By using the dose–response curve for each compound under dark- and irradiated conditions, the effective concentration (EC_{50}) was calculated by fitting the curves to a non-linear regression function with fixed *y* maximum (100 %) and minimum (0 %) (relative cell viability) and a variable Hill slope, which resulted in the simplified two-parameter Hill-slope equation. Photo indices (PI) reported in Table 1 were calculated, for each compound by dividing the EC_{50} value obtained in the dark by the EC_{50} value determined under light irradiation.

Cell uptake

Cell uptake studies for complexes [**1**]Cl_2 and [**2**]Cl_2 were conducted on A549 lung cancer cells. 8×10^5 cells were seeded at t = 0 h in Opti-MEM complete (3 mL) in 6 cm diameter dishes. At t = 24 h cells were treated with solutions of [**1**]Cl_2 and [**2**]Cl_2 to give a final concentration of 20 and 80 µM respectively in a total volume of 6 mL. After 6 h of drug incubation at 37 ºC, the medium was aspirated and the cells were washed twice with 4 mL PBS. Then, the cells were trypsinized (1 mL), suspended with Opti-MEM (3 mL), and centrifuged (1200 rpm, 4 min). After aspiration of the supernatant, the cells were re suspended in PBS (1mL) and counted. After a second centrifugation, the supernatant was discarded and the pellets were resuspended in MilliQ water (154 µL) and 65% HNO_3 (up to 2 mL) for overnight digestion. Then, 1 mL of the solution was diluted with MilliQ water to obtain a final concentration of 5% HNO_3. For ICP-MS measurements, the system was optimized with a ruthenium-platinum solution. The calibration range was from 0 to 25 µg/L, and obtained detection limit for all isotopes was 0.01 µg/L. Silver and Indium were used for internal standard, to correct for sample dependent matrix effects. No reference sample was available; therefore several samples were spiked with a known concentration. The recoveries of the spiked concentrations were all within a 10% deviation. The data from two independent biological replications were used to obtain the uptake values shown in Table 1.

References

1. Howerton, B. S.; Heidary, D. K.; Glazer, E. C., *J. Am. Chem. Soc.* **2012,** *134*, 8324-8327.
2. Wallenstein, J. H.; Sundberg, J.; McKenzie, C. J.; Abrahamsson, M., *Eur. J. Inorg. Chem.* **2016**, n/a-n/a.
3. Reisner, E.; Abikoff, T. C.; Lippard, S. J., *Inorg. Chem.* **2007,** *46*, 10229-10240.
4. Garcìa-Fresnadillo, D.; Georgiadou, Y.; Orellana, G.; Braun, A. M.; Oliveros, E., *Helv. Chim. Acta* **1996,** *79*, 1222-1238.
5. Wang, F.; Xu, J.; Habtemariam, A.; Bella, J.; Sadler, P. J., *J. Am. Chem. Soc.* **2005,** *127*, 17734-17743.
6. Hopkins, S. L.; Siewert, B.; Askes, S. H. C.; Veldhuizen, P.; Zwier, R.; Heger, M.; Bonnet, S., *Photochem. Photobiol. Sci.* **2016,** *15*, 644-653.
7. Vichai, V.; Kirtikara, K., *Nat. Protocols* **2006,** *1*, 1112-1116.
